# Supplementary material for: Treatment with tumor-treating fields (TTFields) suppresses intercellular tunneling nanotube formation in vitro and upregulates immuno-oncologic biomarkers in vivo in malignant mesothelioma
Source: eLife. 2023 Nov 13;12:e85383. doi: 10.7554/eLife.85383 (PMC10642963; doi:10.7554/eLife.85383)
Supplement: Figure 2—source data 1. [file elife-85383-fig2-data1.zip › Annotated Gels/_Annotated Gels_Lou Lab_All_revised.pptx.pdf]

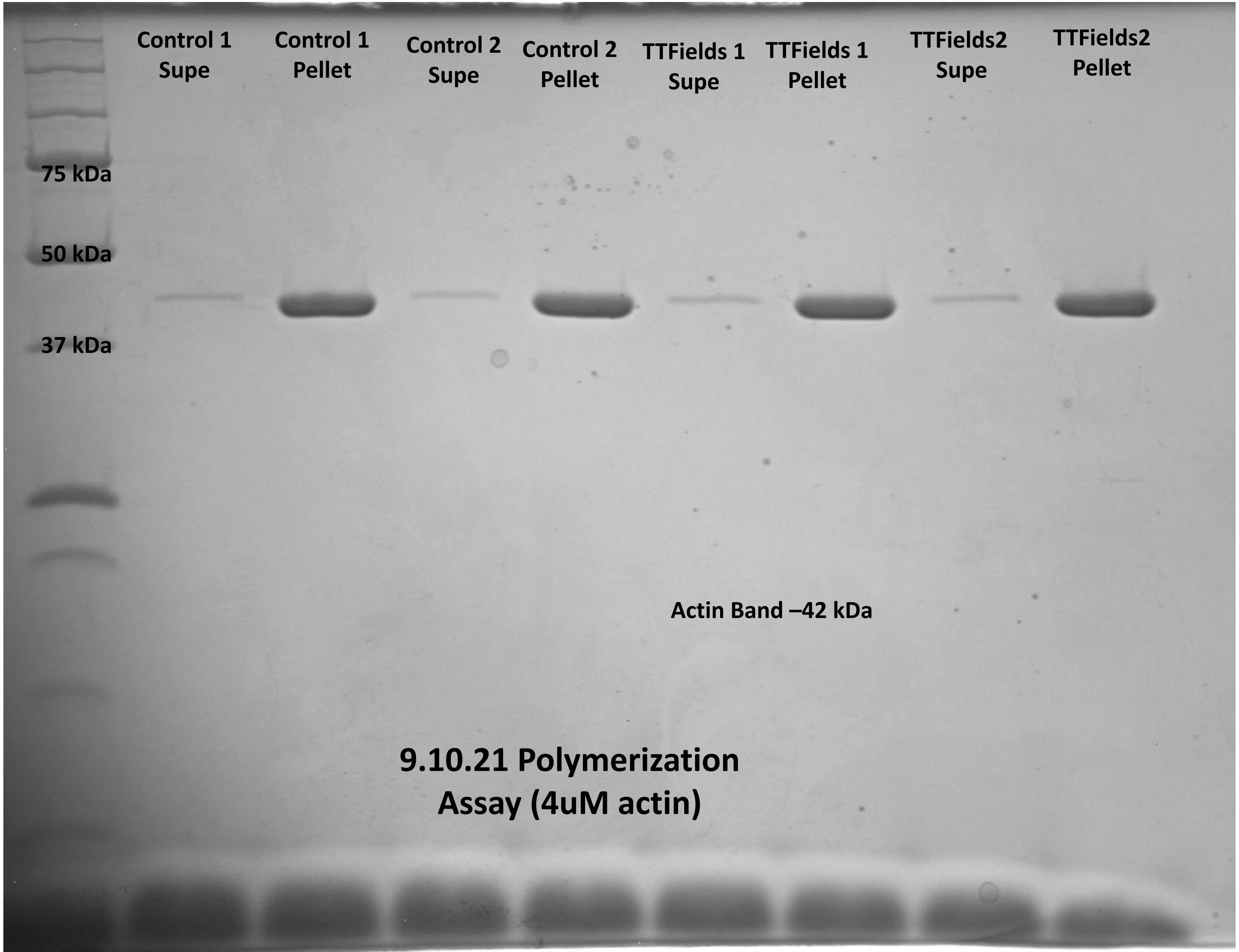
Control 1 Supe
Control 1 Pellet
Control 2 Supe
Control 2 Pellet
TTFields 1 Supe
TTFields 1 Pellet
TTFields2 Supe
TTFields2 Pellet
75 kDa
50 kDa
37 kDa
Actin Band –42 kDa
9.10.21 Polymerization Assay (4uM actin)

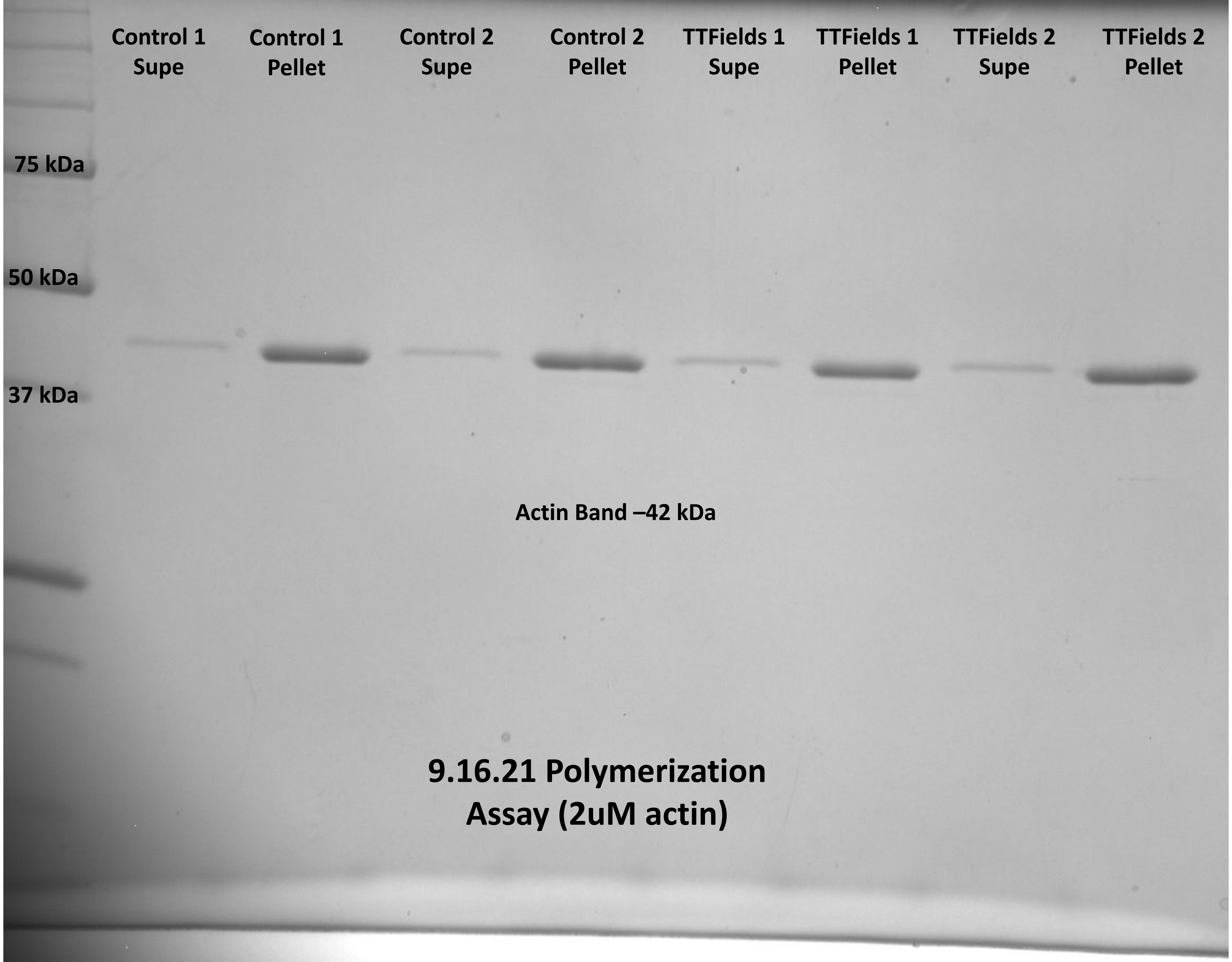
Control 1 Supe
Control 1 Pellet
Control 2 Supe
Control 2 Pellet
TTFields 1 Supe
TTFields 1 Pellet
TTFields 2 Supe
TTFields 2 Pellet
75 kDa
50 kDa
37 kDa
Actin Band –42 kDa
9.16.21 Polymerization Assay (2uM actin)

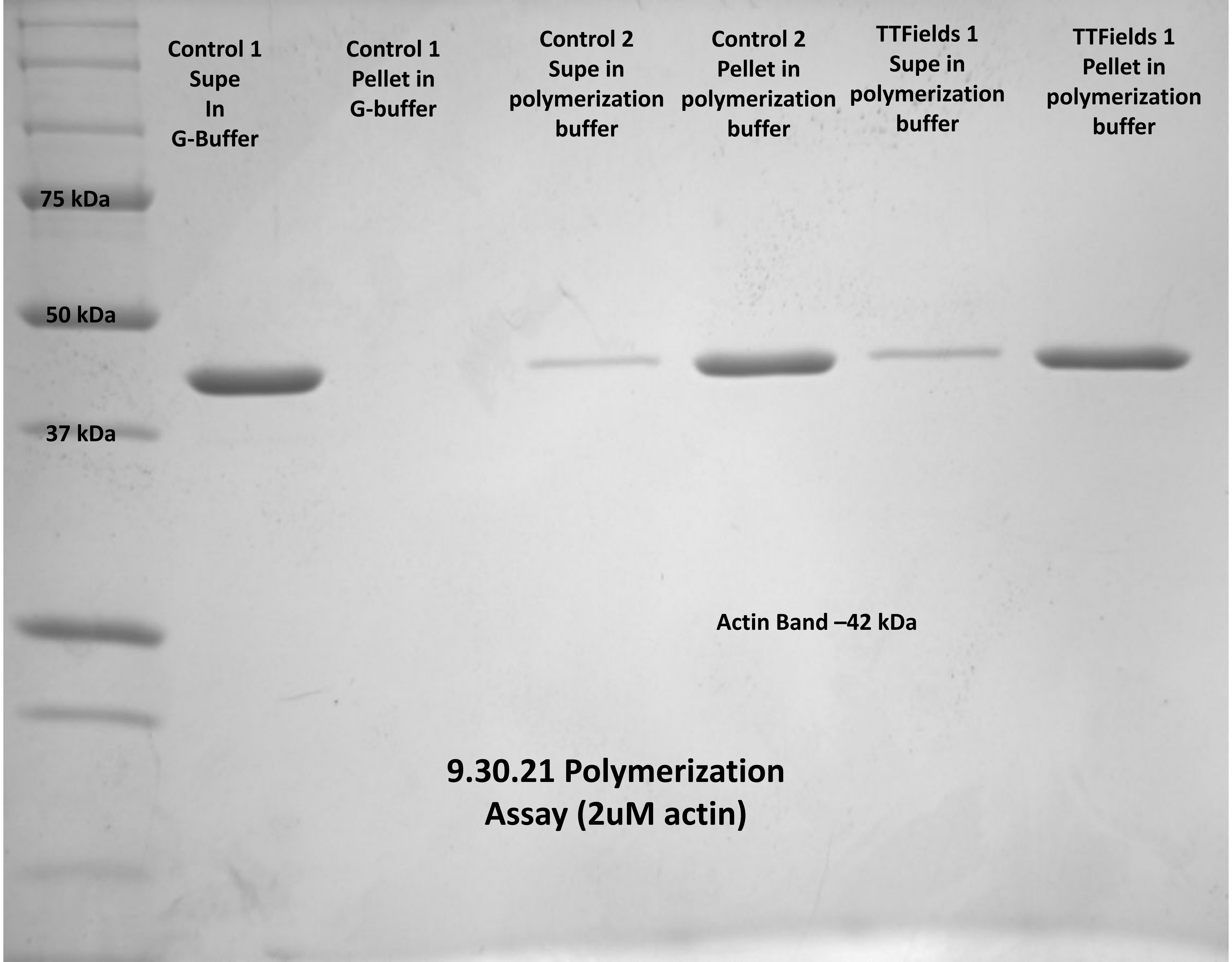
Control 1 Supe In G-Buffer
Control 1 Pellet in G-buffer
Control 2 Supe in polymerization buffer
Control 2 Pellet in polymerization buffer
TTFields 1 Supe in polymerization buffer
TTFields 1 Pellet in polymerization buffer
75 kDa
50 kDa
37 kDa
Actin Band –42 kDa
9.30.21 Polymerization Assay (2uM actin)

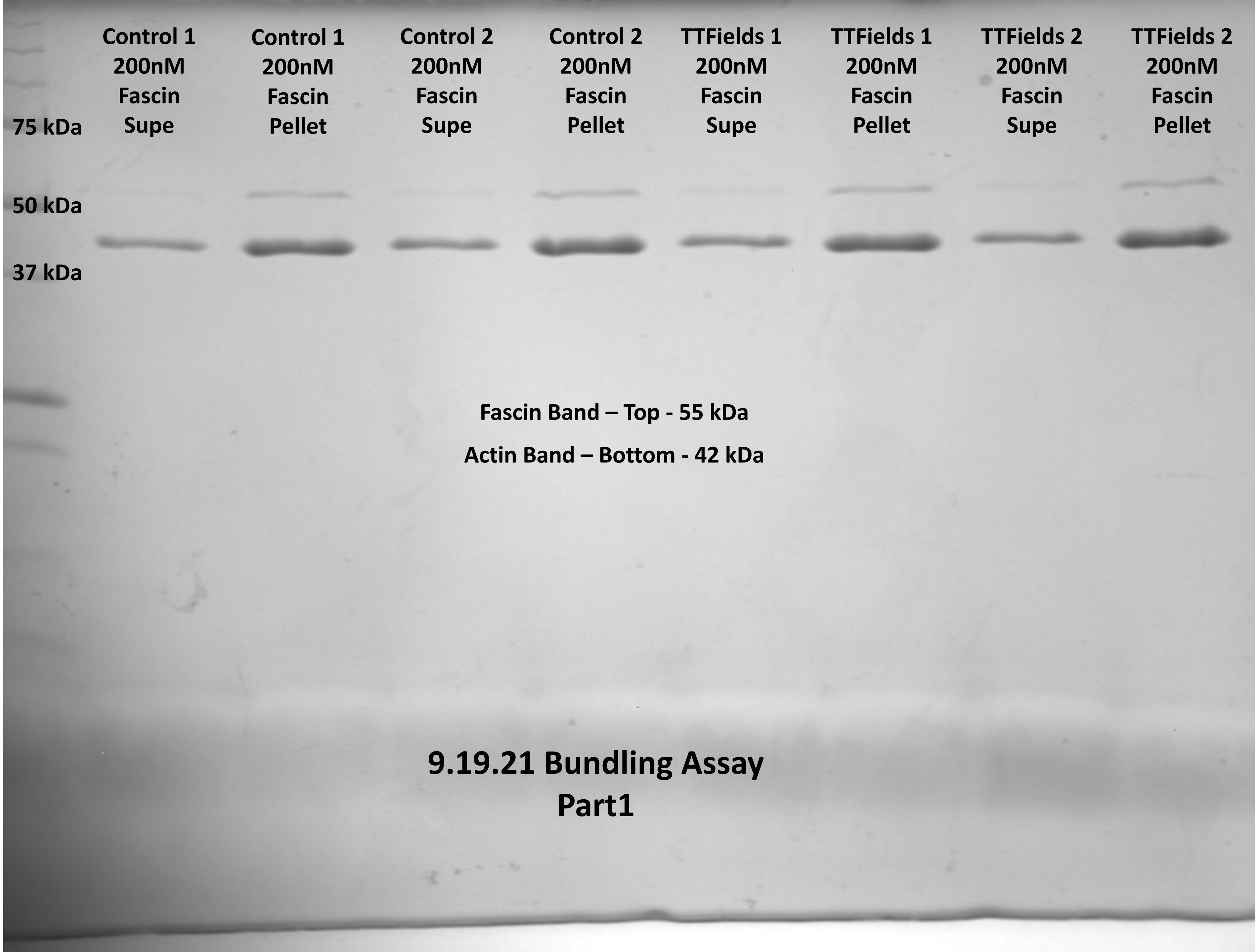
Control 1 200nM Fascin Supe
Control 1 200nM Fascin Pellet
Control 2 200nM Fascin Supe
Control 2 200nM Fascin Pellet
TTFields 1 200nM Fascin Supe
TTFields 1 200nM Fascin Pellet
TTFields 2 200nM Fascin Supe
TTFields 2 200nM Fascin Pellet
75 kDa
50 kDa
37 kDa
Fascin Band – Top - 55 kDa
Actin Band – Bottom - 42 kDa
9.19.21 Bundling Assay
Part1

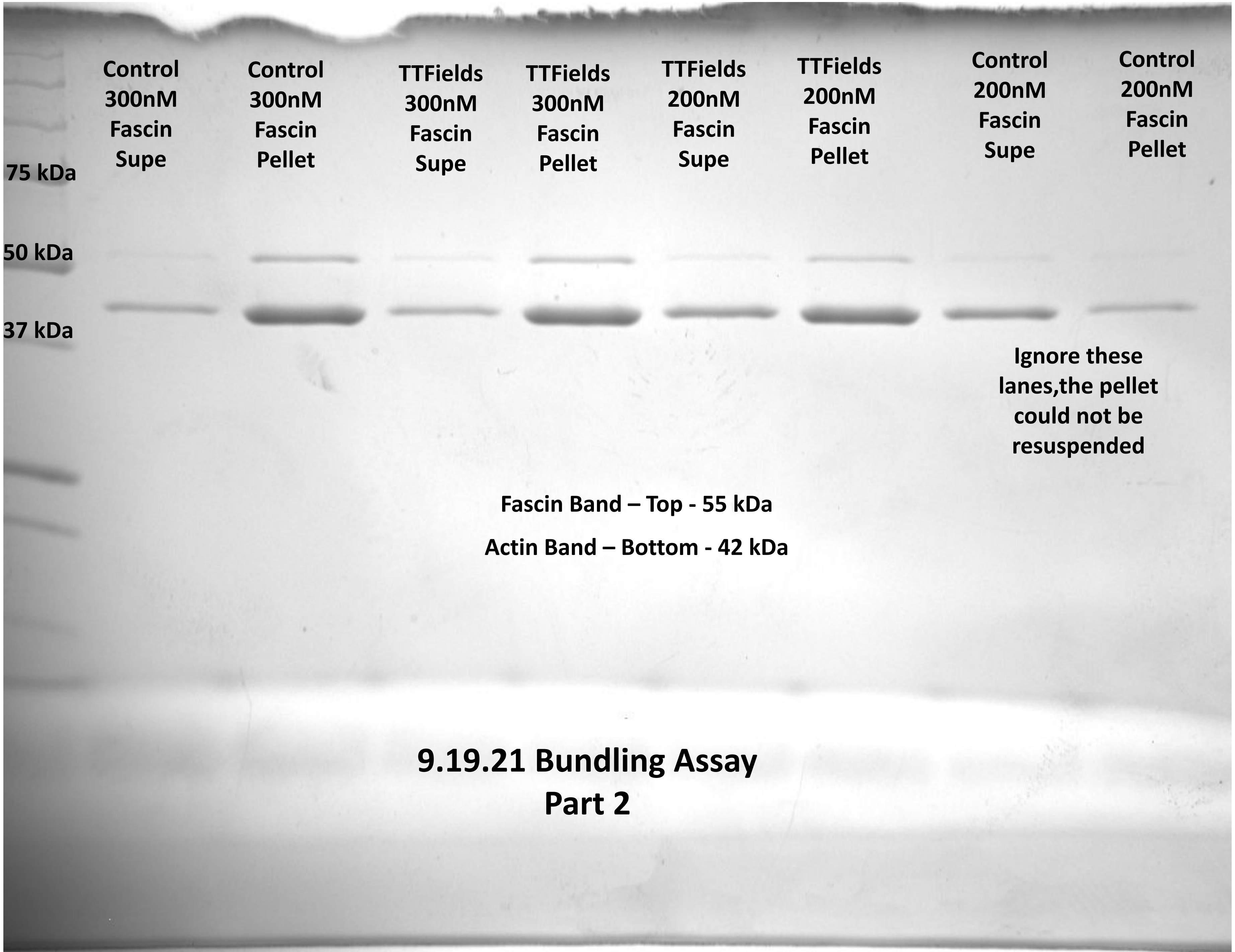
Control 300nM Fascin Supe
Control 300nM Fascin Pellet
TTFields 300nM Fascin Supe
TTFields 300nM Fascin Pellet
TTFields 200nM Fascin Supe
TTFields 200nM Fascin Pellet
Control 200nM Fascin Supe
Control 200nM Fascin Pellet
75 kDa
50 kDa
37 kDa
Ignore these lanes,the pellet could not be resuspended
Fascin Band – Top - 55 kDa
Actin Band – Bottom - 42 kDa
9.19.21 Bundling Assay Part 2

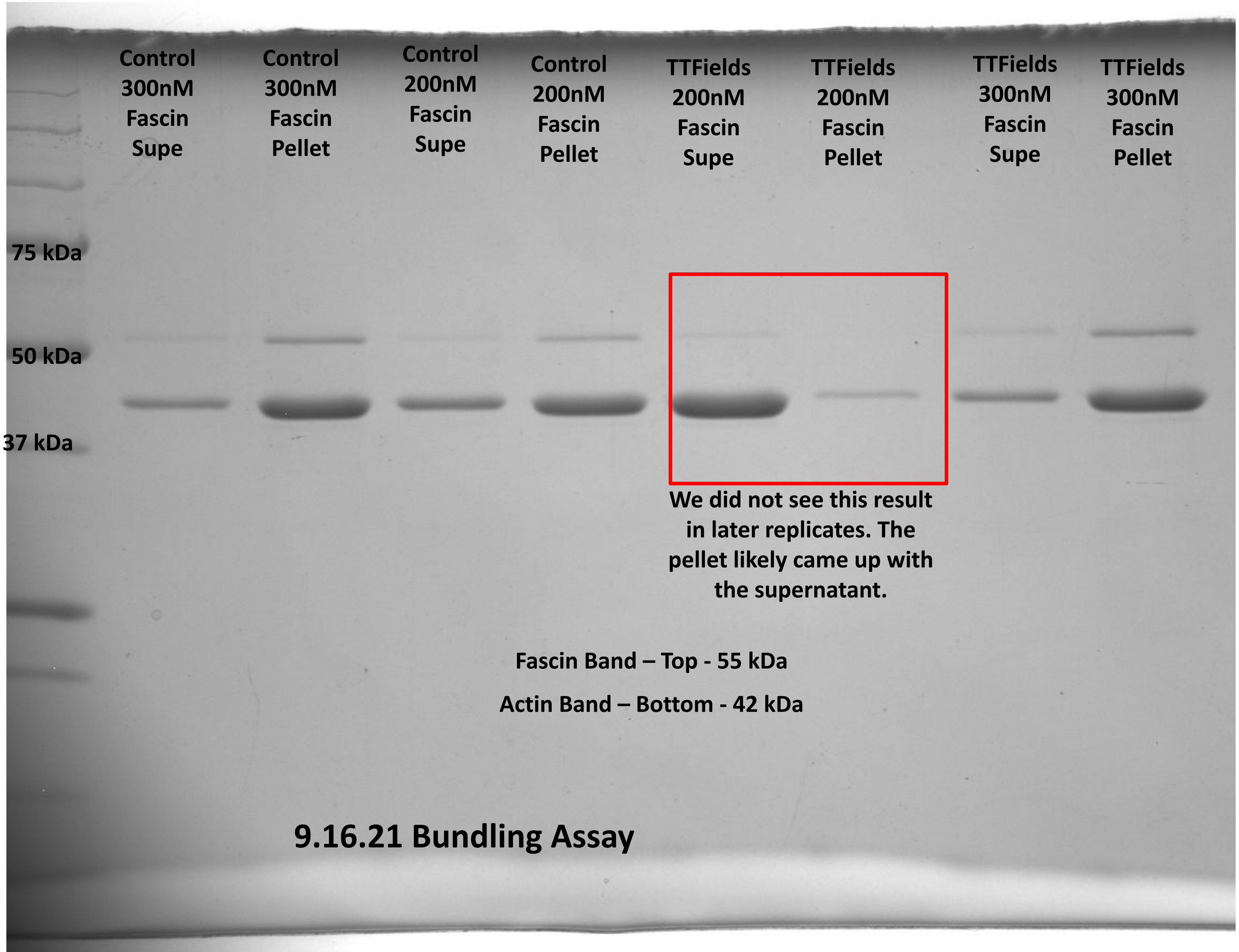
Control 300nM Fascin Supe
Control 300nM Fascin Pellet
Control 200nM Fascin Supe
Control 200nM Fascin Pellet
TTFields 200nM Fascin Supe
TTFields 200nM Fascin Pellet
TTFields 300nM Fascin Supe
TTFields 300nM Fascin Pellet
75 kDa
50 kDa
37 kDa
We did not see this result in later replicates. The pellet likely came up with the supernatant.
Fascin Band – Top - 55 kDa
Actin Band – Bottom - 42 kDa
9.16.21 Bundling Assay

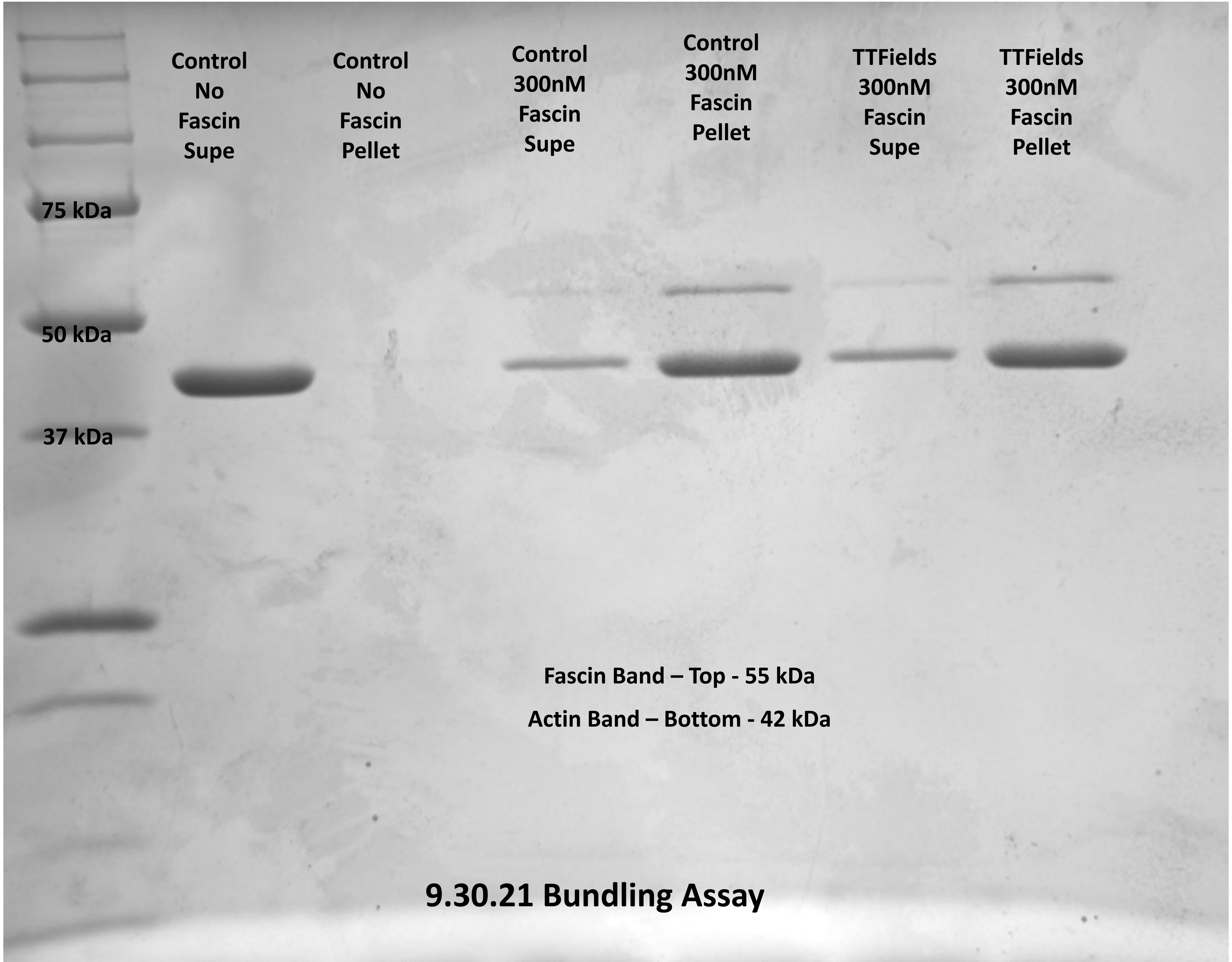
Control No Fascin Supe
Control No Fascin Pellet
Control 300nM Fascin Supe
Control 300nM Fascin Pellet
TTFields 300nM Fascin Supe
TTFields 300nM Fascin Pellet
75 kDa
50 kDa
37 kDa
Fascin Band – Top - 55 kDa
Actin Band – Bottom - 42 kDa
9.30.21 Bundling Assay
