# Supplementary figures and images for: Treatment with tumor-treating fields (TTFields) suppresses intercellular tunneling nanotube formation in vitro and upregulates immuno-oncologic biomarkers in vivo in malignant mesothelioma
Source: eLife. 2023 Nov 13;12:e85383. doi: 10.7554/eLife.85383 (PMC10642963; doi:10.7554/eLife.85383)

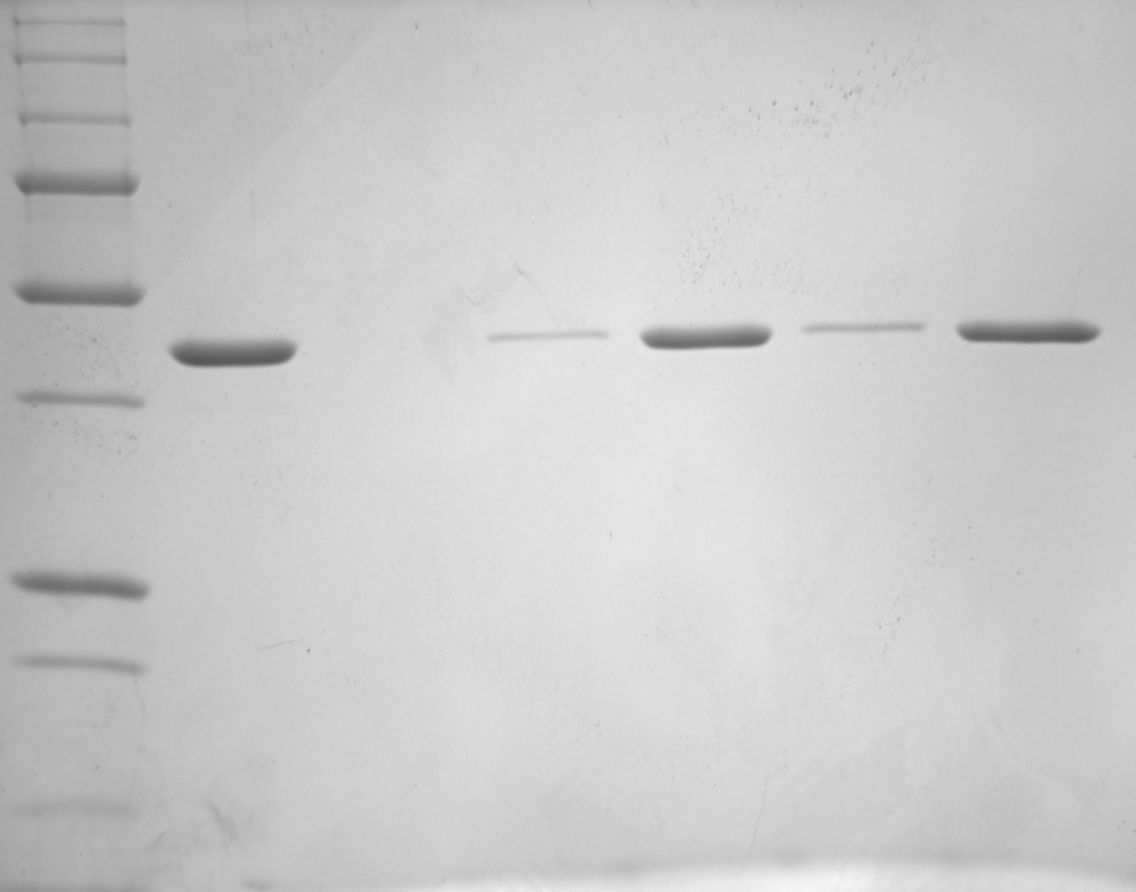

Supplement: Figure 2—source data 1. [file elife-85383-fig2-data1.zip › Raw Gels/9.30.21 Polymerization Sed assay 2uM Actin Lou Lab.tif]

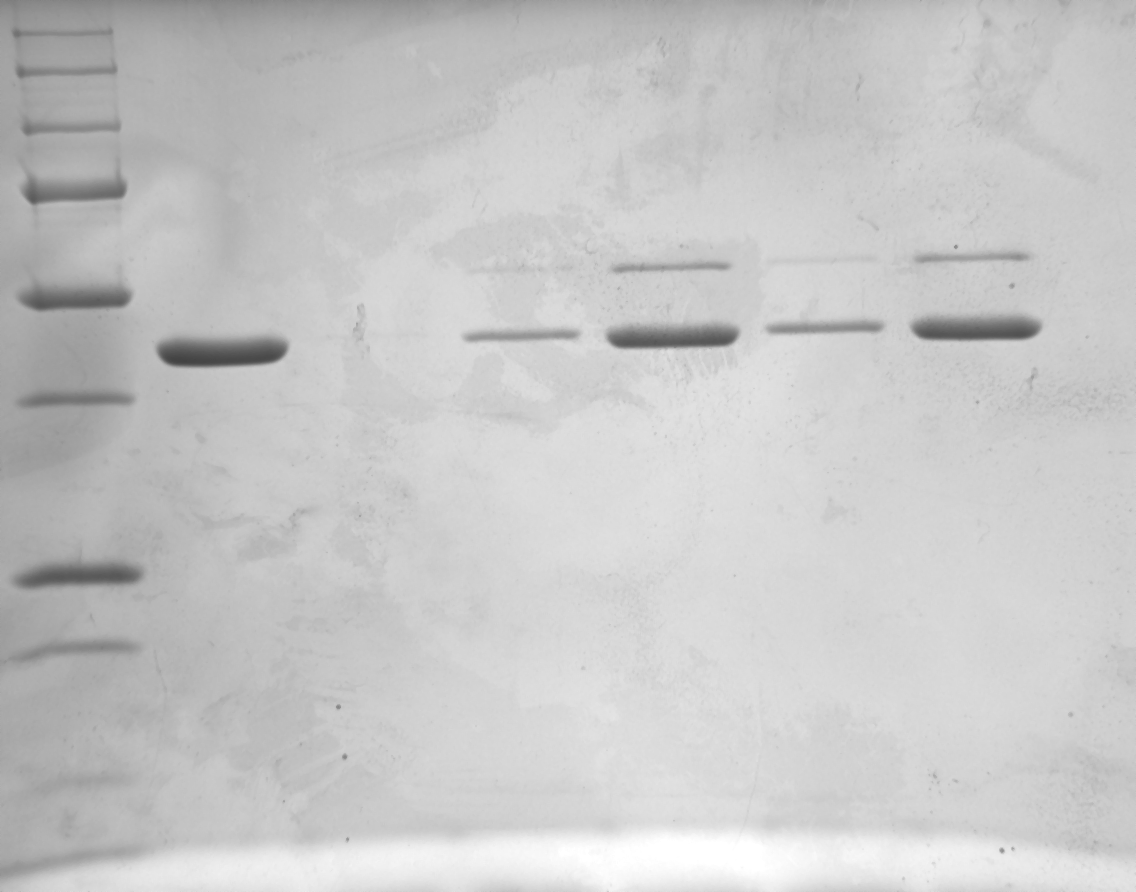

Supplement: Figure 2—source data 1. [file elife-85383-fig2-data1.zip › Raw Gels/9.30.21 Bundling Sed Assay 300 nM Fas Lou Lab.tif]

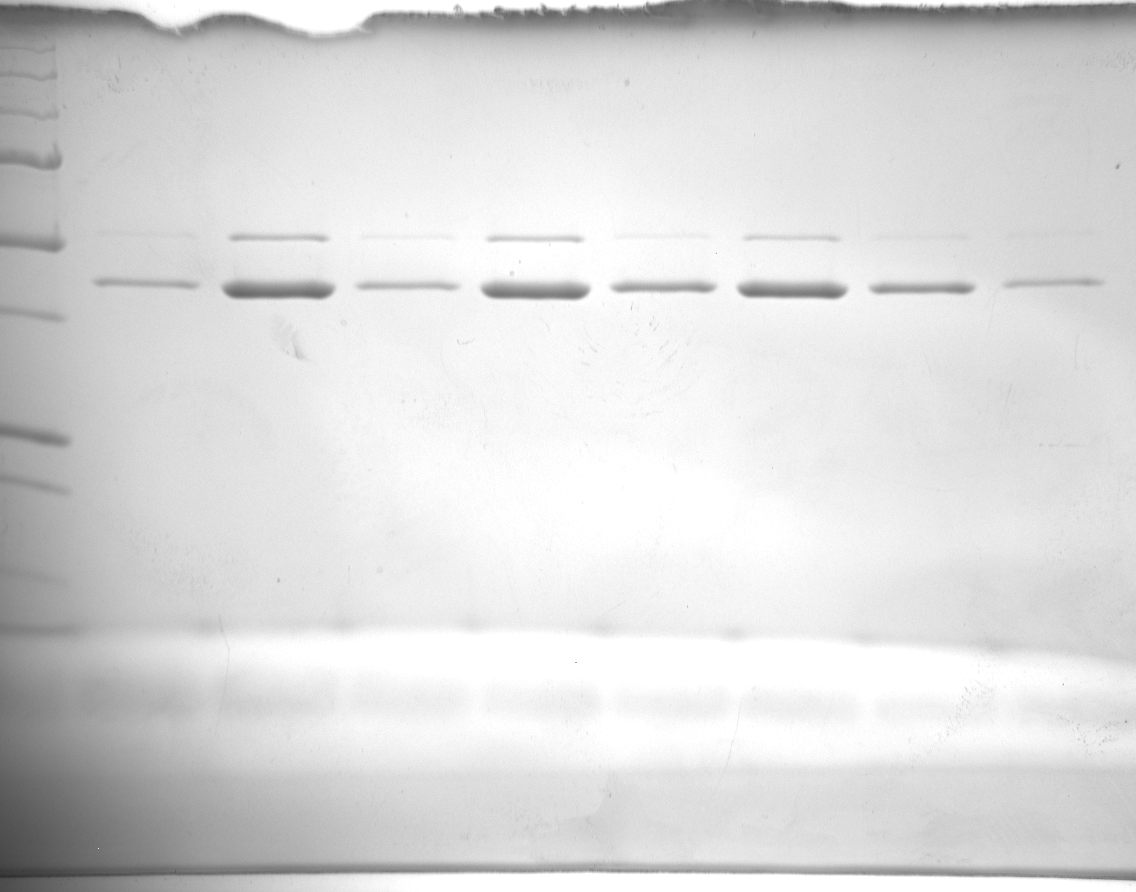

Supplement: Figure 2—source data 1. [file elife-85383-fig2-data1.zip › Raw Gels/Lou_Lab Bundling w300nM.tif]

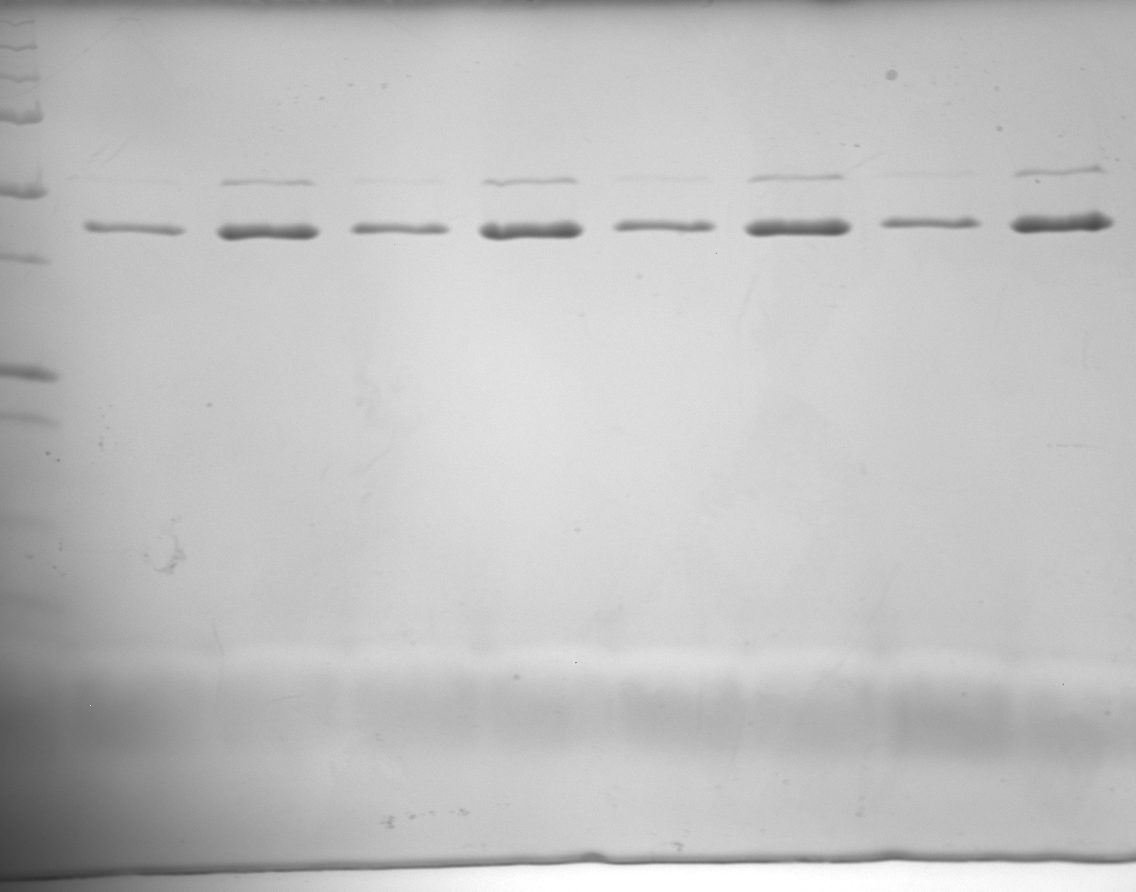

Supplement: Figure 2—source data 1. [file elife-85383-fig2-data1.zip › Raw Gels/Lou_Lab Bundling 200nM.tif]

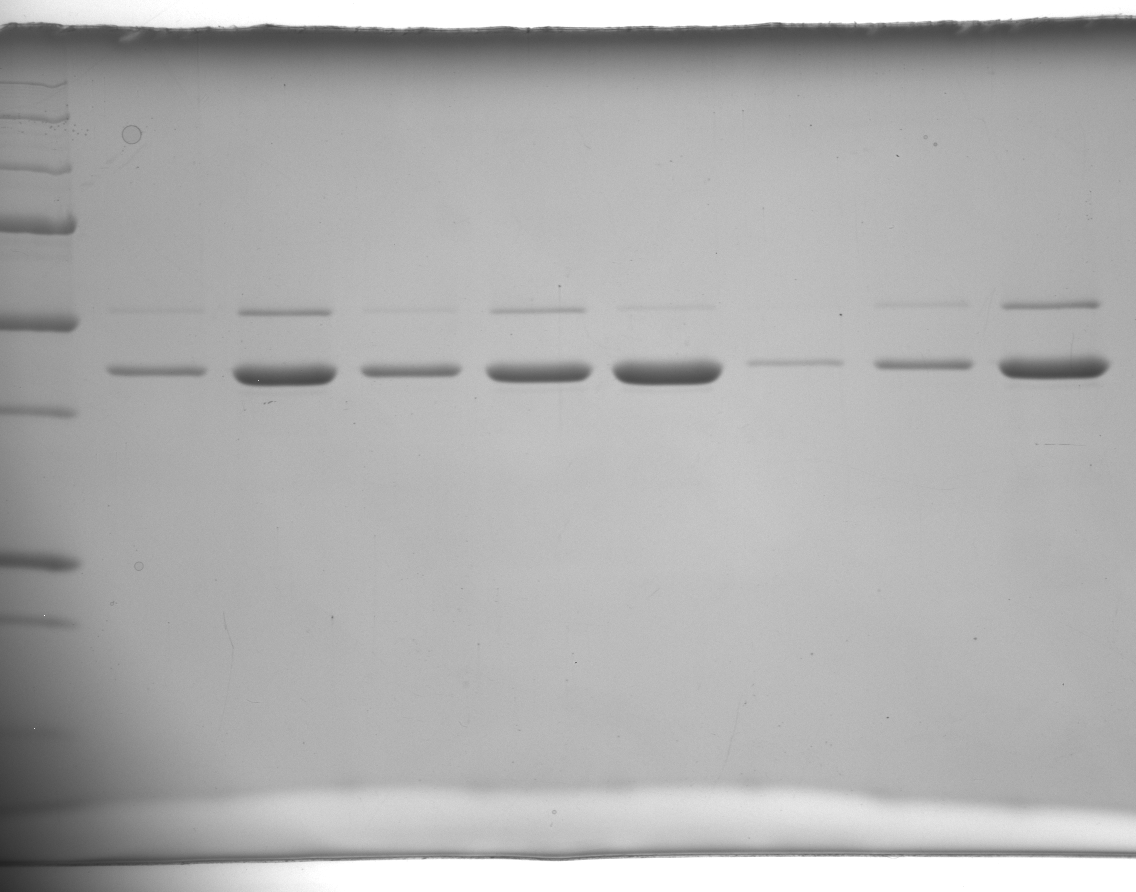

Supplement: Figure 2—source data 1. [file elife-85383-fig2-data1.zip › Raw Gels/9.16.21_Lou_Lab_bundling by fascin.tif]

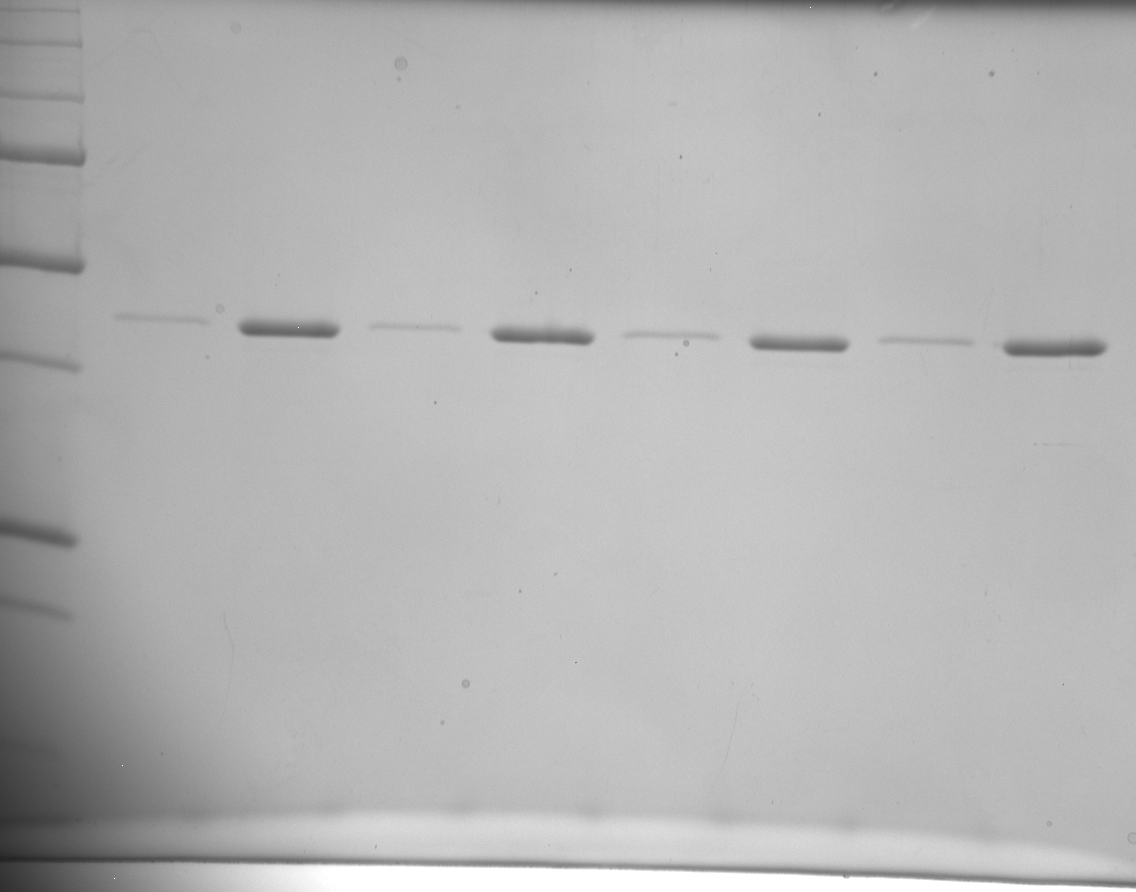

Supplement: Figure 2—source data 1. [file elife-85383-fig2-data1.zip › Raw Gels/9.16.21_Lou_Lab_2uM actin polymerization.tif]

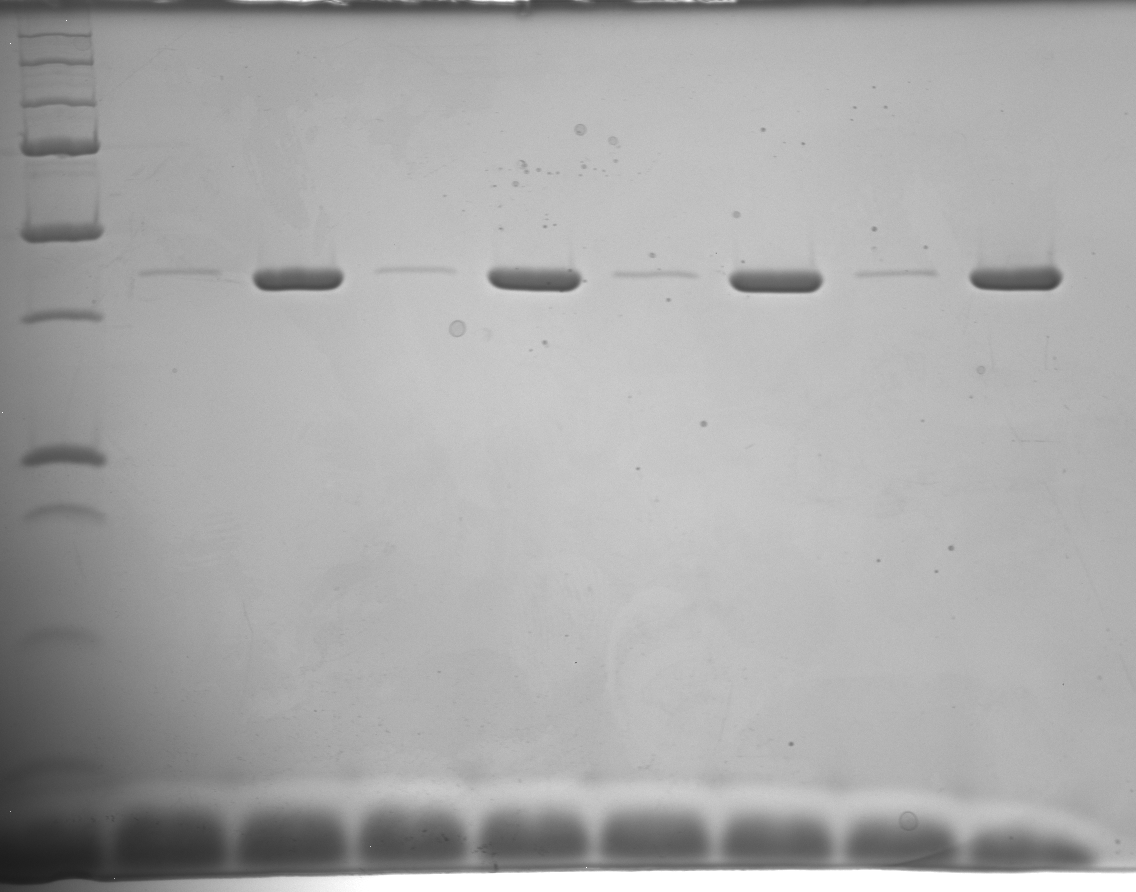

Supplement: Figure 2—source data 1. [file elife-85383-fig2-data1.zip › Raw Gels/9.10.21_Sed_Assay_Lou_Lab.tif]
