# Supplementary material for: Treatment with tumor-treating fields (TTFields) suppresses intercellular tunneling nanotube formation in vitro and upregulates immuno-oncologic biomarkers in vivo in malignant mesothelioma
Source: eLife. 2023 Nov 13;12:e85383. doi: 10.7554/eLife.85383 (PMC10642963; doi:10.7554/eLife.85383)
Supplement: Supplementary file 1. [file elife-85383-supp1.pdf]

Supplementary Materials and Methods:

inovitro and inovitro Live TTFields treatment:

The inovitro^{TM} device, provided by Novocure, Ltd (Haifa, Israel), was used to apply continuous bidirectional TTFields treatment to cells. One day prior to treatment with TTFields, 22-mm plastic cell-culture treated coverslips (Thermo Fisher Scientific Nunc Thermanox, Waltham, MA, USA) were placed inside sterile ceramic dishes. MSTO-211H cells (40,000) in 2 ml of growth media were plated onto the coverslips, and the dishes were placed in a base plate in a humidified incubator at 37°C with 5% carbon dioxide overnight. To apply TTFields to the cells, the ceramic dishes were connected to an inovitro Generator Box. inovitro software controls and monitors the electrical resistance, voltage, and current in real time, while the temperature in the incubator is directly correlated with the intensity of the electric field. The temperature was set at 32°C to deliver an intensity of 0.5 V/cm and at 26.5°C for an intensity of 1.0 V/cm[20]. Additionally, the frequency of the electric field was set at 200 kHz for all conditions in both cell lines, barring any initial frequency testing and cell viability assessment. All intensity values were expressed in root mean square (RMS) values to illustrate the conventional depiction of alternating current measurements in physics fields. The treated group was exposed to TTFields for 72 hours in both 0.5 V/cm and 1.0 V/cm experiments. For the 1.0 V/cm experiments, the TTFields were shut off at 72 hours, and the cells were incubated for another 24 hours to assess recovery of TNTs. Cells in the control group were not treated with TTFields and were plated as described above and placed in an incubator at 37°C with 5% carbon dioxide for the duration of the experiment. The low-density experiments were run as described above with the exception that only 10,000 cells were plated onto a coverslip, and TTFields application followed 3 hours later.

The inovitro Live^{TM} device, provided by Novocure, Ltd (Haifa Israel), was used to apply continuous unidirectional or bidirectional TTFields exposure to cells. One day prior to treatment, 40,000 MSTO-211H cells were plated onto a 35 mm high wall, glass bottom dish (Ibidi, Gräfelfing, Germany), and allowed to adhere overnight. For the unidirectional and bidirectional experiments, the glass bottom dish was coated with Poly-D Lysine (Millipore Sigma, Burlington, MA) at a concentration of 1mg/µm for 1 hour then dried for 2 hours prior to plating. The next day, an inovitro Live insert was positioned in the 35 mm dish, and placed in the microscope chamber. The plate was connected to an inovitro Live cable, and a heating element was added on top of the dish cover to minimize condensation from heat generated by TTFields. The cable was then connected to an inovitro Live Generator, and the software controlled the delivery of an electric field in either one (unidirectional) or two (bidirectional) directions at an intensity of 1.0 V/cm and either 150 or 200 kHz. Media was changed every 24 hours, during which TTFields were paused and then resumed once the cells were placed back into the incubator. The cells for the control group were plated as described above and placed in the microscope chamber at 37°C, without TTFields, for the duration of the experiment. Seven Fields of View (FOV) were selected every 24 hours, up to 72 hours and both cell proliferation and TNT formation were quantified. As an additional experimental arm, MSTO-211H cells were also treated with cisplatin (160 nM) and pemetrexed (24 nM) in conjunction with TTFields application using pre-treated ibidi plates. During these experiments, images were acquired for 4 hours at 2min/frame, and this process repeated every 24 hours, up to 72 hours total. Both cell proliferation and TNT formation were subsequently quantified as described above.

Actin and Fascin Purification

Actin was purified from chicken skeletal muscle by one cycle of polymerization and depolymerization using standard protocols in the field (Spudich et al.). It was then filtered on Sephacryl S-300 resin (GE Healthcare) in G-buffer (2 mM Tris (pH 8.0), 0.2 mM ATP, 0.5 mM DTT, 0.1 mM $CaCl_2$) to obtain actin monomers, and stored at 4°C. Human fascin-1 was expressed with an N-terminal glutathione s-transferase (GST) tag and a TEV cleavage recognition sequence from the pGV67 plasmid in BL21 DE3pLysS competent cells. Transformants were grown in 1 L of LB broth, induced at OD_{600} ~0.6 with 0.5 mM IPTG, and shaken overnight (200 rpm, 17°C). To purify fascin, cell pellets were resuspended in lysis buffer (50 mM Tris, pH 8.0, 500 mM NaCl, 1 mM DTT) and sonicated. Lysed cells were centrifuged (~30,000 x g, 4°C) for 40 minutes to isolate the soluble cell components. Samples were rotated with glutathione agarose resin (pH 8.0) for 1 hour at 4°C, washed, and eluted (50 mM Tris, pH 8.0, 100 mM NaCl, 1mM DTT, 100 mM glutathione). Eluted fractions were incubated with TEV protease (1.6 μM) for GST tag cleavage and dialyzed into glutathione-free buffer overnight. To remove GST contaminants and TEV protease, samples were filtered through glutathione resin followed by amylose resin. Collected flow throughs were concentrated using centrifugal filters (Millipore Sigma Amicon, MWCO 30K). Samples were frozen in liquid nitrogen and stored at -80°C.

Actin Polymerization and Bundling Sedimentation Assays

Actin was polymerized at 37°C in KMEI buffer (50 mM KCl, 1 mM $MgCl_2$, 1 mM EGTA, 10 mM Imidazole pH 7.0) for 1 hour with and without 1.0 V/cm inovitro device TTFields treatment. Samples were centrifuged at 100,000 x g for 30 minutes at 4°C to separate filaments and monomers. Supernatant and pellet fractions were analyzed via SDS-PAGE (12% acrylamide).

Gels were then stained with Coomassie Blue for 1 hour and destained for at least 6 hours (10% ethanol, 7.5% acetic acid). Band intensities were quantified via densitometry using Fiji-ImageJ. For bundling, actin (15 µM) was first polymerized for 1 hour at 37°C in KMEI buffer. The assembled filaments were diluted to 3 µM and added to a solution with fascin (300 nM). After 1 hour with and without 1.0 V/cm TTFields treatment, samples were centrifuged at 10,000 x g for 30 minutes at 4°C to pellet bundled actin. SDS-PAGE and band quantification were carried out as described previously.

Microscopy imaging of intact tumors from our animal model of MPM

Eight BALB/c mice were injected with murine AB mesothelioma cells, and then treated with either TTFields or heat sham (negative control).Tumors were excised and sectioned into 15 um sections. These sections were mounted on coverslips, deparaffinized, and stained with a nuclear dye (Sytox Green 488, ThermofisherScientific, USA, S7020) and a phalloidin stain (Alexa Fluor 647, ThermofisherScientific, USA, A22287) following permeabilization with 0.1% Triton-X. They were then mounted with Prolong Gold Antifade reagent (ThermofisherScientific, USA, P36930) and covered with 1.5 thickness coverslips. Fluorescence images were acquired by using an inverted Nikon Ti- Emicroscope (NIKON A1R SI, Tokyo, Japan) and an MCL NanoDrive Piezo Z Drive stage (Mad City Labs Inc. Wisconsin, United States) through a 60x oil immersion objective lens (NA=1.4, Plan Apo lambda correction collar, Tokyo, Japan). Samples were excited with 488 nm laser power set to 3.5% and a 638 nm laser with power set to 14.2%. Lasers were scanned with Galvano mirrors at a scanning speed of 0.25 with a zoom setting of 2.392 and a line average of 8. Pinhole diameter was set to 38.31 um and emitted light was passed through a 408/488/561/640 dichroic mirror. Emitted light was then detected by DU4 GaAsP detectors with gain settings of 54 for the blue light detector and 96 for the far-red light detector. Filter cubes

from the Chroma series were used for both dyes (99022 and 99023) for Sytox 488 and Alexa Fluor 647, respectively. Images were captured on a Hamamatsu FLASH 4 camera (Hamamatsu Photonics, Hamamatsu City, Japan) with voxel dimensions of 0.108 μm in XY and 0.222 μm in Z with PMT confocal detectors and using NIKON A1 Elements software (NIKON, Melville, New York, United States) for acquisition. To calculate step size of z stacks and optimal imaging resolution, Nyquist sampling was performed. Images were processed with iterative prediction advanced denoising followed by 3D automatic deconvolution with a theoretical point spread function (based on emission wavelengths of fluorophores used), automatic background subtraction, and spherical aberration correction. Z stacks were then rendered and animated using Imaris (Oxford Instruments, Beijing, China, version 5.42.03).
